# Ionizing Radiation and Its Effects on Thermoplastic Polymers: An Overview

**DOI:** 10.3390/polym17081110

**Published:** 2025-04-19

**Authors:** Ary Machado de Azevedo, Pedro Henrique Poubel Mendonça da Silveira, Thomaz Jacintho Lopes, Odilon Leite Barbosa da Costa, Sergio Neves Monteiro, Valdir Florêncio Veiga-Júnior, Paulo Cezar Rocha Silveira, Domingos D’Oliveira Cardoso, André Ben-Hur da Silva Figueiredo

**Affiliations:** 1 Department of Materials Science, Military Institute of Engineering-IME, Praça General Tibúrcio, 80, Urca, Rio de Janeiro 22290-270, Brazil; pedroo.poubel@gmail.com (P.H.P.M.d.S.);; 2 Department of Chemistry, Military Institute of Engineering-IME, Praça General Tibúrcio, 80, Urca, Rio de Janeiro 22290-270, Brazil; valdir.veiga@gmail.com; 3 Department of Nuclear Engineering, Military Institute of Engineering-IME, Praça General Tibúrcio, 80, Urca, Rio de Janeiro 22290-270, Brazil; silveira.paulo@ime.eb.br (P.C.R.S.);

**Keywords:** ionizing radiation, thermoplastic polymers, polymeric degradation, molecular crosslinking, structural modification, industrial applications

## Abstract

This article explores the foundational principles of ionizing radiation and provides a comprehensive overview of its impact on thermoplastic polymers. Ionizing radiation, encompassing gamma rays, X-rays, and electron beams, has been extensively studied due to its capacity to alter the molecular structure of polymers. These changes enable advancements in various applications by promoting molecular crosslinking, controlled degradation, molecular grafting, and crystallinity adjustments. The article delves into the fundamental mechanisms of radiation thermoplastic polymer interactions, including ionization, electronic excitation, and free radical formation. It highlights how these processes lead to structural transformations that enhance the physical, thermal, and mechanical properties of thermoplastic polymers. Factors such as radiation type, absorbed doses, temperature, and environmental conditions are discussed in the context of their role in controlling these modifications. Key practical applications are identified across fields such as medicine, food packaging, aerospace, and industry. Examples include the production of sterilizable medical devices, enhanced food packaging for longer shelf life, and radiation-resistant materials for the aerospace and nuclear sectors. Despite its many advantages, the article also emphasizes challenges such as process variability, polymer sensitivity to radiation, and standardization difficulties. The review underscores emerging research directions, including optimizing irradiation parameters and integrating advanced characterization techniques like Fourier Transform Infrared Spectroscopy (FT-IR) and X-ray diffraction (XRD). The development of new polymer blends and composites, designed for irradiation-induced property enhancement, represents a promising area of innovation.

## 1. Introduction

Polymer irradiation represents an interdisciplinary field of study with historical roots in the advancements of the nuclear industry, initiated in the 1940s [1,2]. This process involves the use of different forms of radiation, including electromagnetic spectra, such as gamma rays and X-rays, as well as atomic and subatomic particles, such as electrons and ions. When polymers are subjected to these radiations, significant transformations occur in their molecular structures, altering their physical, chemical, and mechanical properties [3,4,5]. These modifications enable materials to acquire new characteristics, making them more suitable for specific applications in industries, medicine, and various technological areas [6,7].

Irradiated polymers are essential in many fields because of the versatility of the effects induced in the material. Irradiation can promote a process called crosslinking between polymer chains, which confers greater thermal, chemical, and mechanical resistance to the material [8,9]. However, under certain conditions, irradiation can induce controlled degradation of polymer chains, resulting in lighter, soluble, or biodegradable materials. Moreover, the monomer can be grafted onto irradiated polymers, creating hybrid materials with unique properties, such as increased biological compatibility or novel electrical and optical functionalities [10,11].

The scientific and industrial interest in polymer irradiation has grown exponentially with time because of its wide range of practical applications. In the medical sector, for example, irradiated polymers are used in the manufacturing of sterile devices, such as syringes, catheters, and blood bags, due to their ability to withstand radiation sterilization processes without compromising their properties [12,13,14,15]. In the food industry, irradiation is applied in polymers to create packaging that extends product shelf life, protecting against microbiological contamination and environmental impacts. Furthermore, some polymers are more sensitive to radiation, limiting their applications in certain contexts [16,17,18]. In engineering, polymers modified by radiation-induced crosslinking are widely used in the manufacture of electrical cables, insulators, and high-performance components that must withstand extreme conditions [19,20,21,22].

The effects of radiation on polymers depend primarily on the polymer chain structure, as well as other factors such as the type of radiation, the absorbed doses, the dose rate, the temperature during the process, and the presence of oxygen or other gases in the environment [23]. Regarding ionizing radiation, gamma rays from sources such as cobalt-60 and accelerated electron beams are the most common techniques because of their ability to penetrate materials. However, each method presents advantages and limitations that must be carefully evaluated depending on the desired application [24].

**Figure 1:**
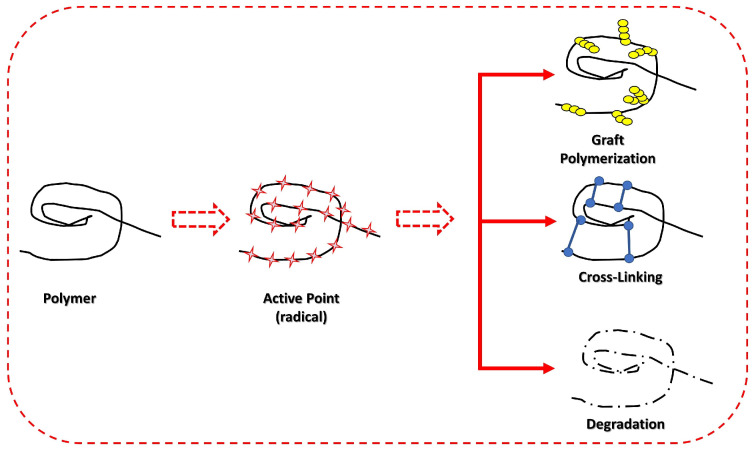
Modifications regarding polymers caused by ionizing irradiation.

From a mechanistic perspective, the interaction of radiation with polymers involves the excitation or ionization of atoms in the polymer chain. These initial events lead to the formation of free radicals, which are highly reactive species. Radicals can interact with each other, resulting in crosslinking formation, or react with environmental molecules, such as oxygen, which can lead to oxidation and degradation [19,25]. Therefore, the choice regarding experimental conditions is crucial to control the outcomes of the irradiation process. The scheme illustrating the effects of irradiation in polymers is presented in Figure 1.

Despite its many benefits, polymer irradiation presents significant challenges. One of the main obstacles is the precise control of structural changes and property modifications in polymers. Small variations in absorbed doses or environmental conditions can lead to unexpected results, complicating the standardization of processes on an industrial scale. Furthermore, some polymers are more sensitive to radiation, limiting their applications in certain contexts.

**Figure 2:**
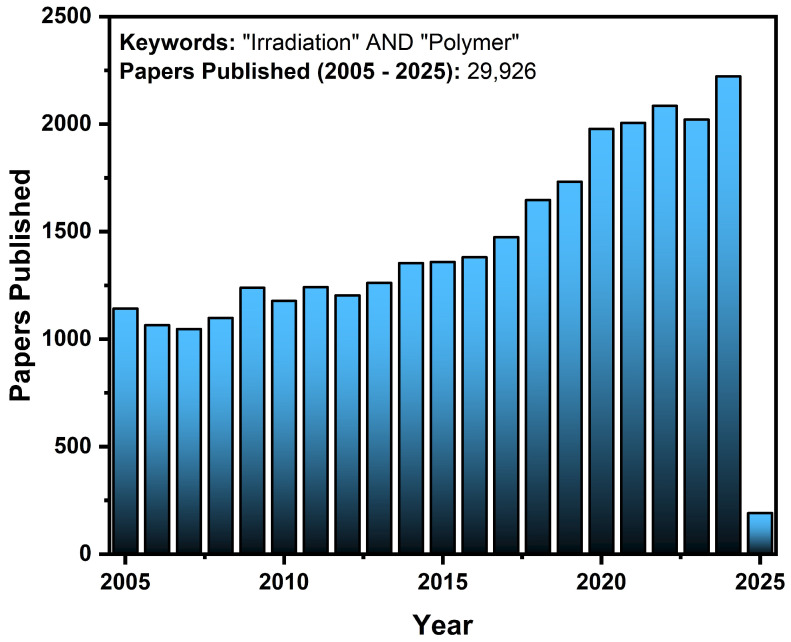
Numbers of papers published between 2005 and 15 January 2025.

The scientific research in the field of thermoplastic polymer irradiation has advanced significantly, exploring new possibilities and addressing complex challenges. Since the first reports on linear polymer irradiation, published by Little in 1952 [26], a growing number of studies have investigated the applications of this technique in various contexts. Data obtained from a bibliographic survey conducted on the SCOPUS platform, presented in Figure 2, highlight this progress: between 2005 and 2025, a total of 29,926 publications were identified, demonstrating a significant increase in academic and industrial interest in this area.

Building on this promising scenario, this article aims to review the literature on thermoplastic polymer irradiation, presenting the state of the art and highlighting the most recent research on the topic. The purpose of this review is to understand the effects of ionizing radiation on polymers, to summarize the dosimetry result, to analyze the methodologies and results achieved over time, and to explore emerging applications of these materials. Through this approach, it is expected not only to advance scientific knowledge but also to strengthen the connection between academic research and industrial demands, fostering technological innovations that promote more sustainable and relevant solutions for contemporary society.

This overview is only about thermoplastic polymers, understanding that thermosetting polymers, such as epoxy, phenolics, and polyester, present similar reactions, like a crosslink [27,28,29], but may present specific reactions when subjected to ionizing radiation. Just like thermoplastic polymers, thermosetting polymers deserve a review, which is the objective of an ongoing paper.

## 2. Theoretical Basis

### 2.1. Basic Effects of Polymer Irradiation

Degradations involve mechanisms at the molecular level, such as [6,30].

- Excitation;
- Charge recombination;
- Trapped electrons and holes;
- Charge transfer;
- Formation of radicals.

#### 2.1.1. Excitation

Excitation is the process by which atoms or molecules absorb enough energy to raise their electrons to higher energy levels without ejecting these electrons from the atom or molecule. This means that the electrons remain bound but occupy energy states higher than the ground state. Excitation can be induced by different energy sources, such as non-ionizing and ionizing electromagnetic radiation, as well as collisions with charged particles. Equation (1) illustrates the excitation process [30].

(1) $$M\overset{Radiation}{\to }M^{*}$$

In polymeric materials, excitation plays an important role in processes involving radiation interactions. When polymers are exposed to ionizing radiation, their molecules cannot only be ionized but also excited, leading to temporary changes in electronic configuration. These changes may result in the formation of excited states that, in some cases, relax by emitting energy as light through the phenomena of fluorescence or phosphorescence. They may also transfer energy to other atoms or molecules, initiating chemical reactions.

Excitation can also be the initial step for creating radical cations or for the breaking of polymer chains in materials, leading to effects such as crosslinking or degradation [31,32].

#### 2.1.2. Charge Recombination

The ionized electrons of the irradiated molecules are subjected to an electric field from the positive charges that form around them. The charge recombination process is common in materials during irradiation or after it has ended [33].

In this ionization neutralization process, a portion of the energy (10–15 eV) is recovered, generating excited molecules with energy greater than any bonding energy. It should be noted that bonds in organic molecules range from 2.3 to 8.9 eV [6,34].

These highly excited molecules can easily break apart to form radicals, as schematically represented in Equation (2) [30].

(2) $$M^{•+}+e^{-}\to M^{**}$$

#### 2.1.3. Trapped Electrons and Holes

Electrons and holes can become trapped within the irradiated material. In organic molecules, the yield of trapped electrons is lower in nonpolar environments compared to polar environments, ranging from 0.1 to 3 electrons per 100 eV [6].

This yield is considerably reduced in crystals compared to that in glasses. The lifetime of trapped electrons depends on parameters such as temperature, the presence of impurities, or polar groups [35].

In organic molecules with long-lived excited states, the migration of excitons over long distances is allowed, thus increasing the production of free charges. This is crucial for various optoelectronic applications, such as photovoltaic cells, photocatalytic reactions, and molecular sensing [36].

#### 2.1.4. Energy Transfer

Intramolecular energy transfer plays an important role in radiolysis. The migration of energy through a molecule and its localization in certain molecular groups largely determine the regularities of the process [35].

In most polymers, electronic excitation energy can be transferred from a donor to an acceptor through three nonradiative mechanisms: resonance energy transfer, induction resonance transfer, and molecular exciton transfer [6,35].

Resonance energy transfer occurs when there is an exchange of electrons between an excited molecule and another molecule in its ground state. It is primarily a triplet-to-triplet transfer, requiring the overlap of molecular wave functions and occurring over short distances, approximately 10 Å [6,37,38].

Induction resonance transfer occurs through electromagnetic interactions between dipoles. It is a singlet-to-singlet transfer and occurs over distances of up to 50 Å. For this resonant transfer, the emission spectra of the donor and acceptor must strictly overlap [6,39].

Molecular exciton transfer can be described as the propagation of the exciton, which involves energy transfer between the electronic excitation levels of interacting molecules, where the energy level of the electron cannot be localized in any of these molecules individually. The mobility of the exciton and its probability of being captured by traps are the main characteristics of this mechanism [40,41,42].

Excitons are bound states of an electron and a hole, in the absence of an electron, that are attracted to each other by the Coulomb force. These quasiparticles are formed when a molecule absorbs light or otherwise gains energy. Specifically, when a molecule absorbs a photon, it excites an electron to a higher energy state, leaving behind a hole in the lower energy state. This process results in the creation of an exciton, which is a neutral entity. The exciton then moves through the material as a whole, carrying the absorbed energy with it [43,44].

#### 2.1.5. Formation of Free Radicals

Free radicals are molecules or atoms that have the capacity to form new bonds due to the presence of unpaired electrons. They can be generated by ionization or excitation of a molecule [6,45]. In polymers, which are composed of macromolecules composed of repeating units called monomers, the formation of free radicals is directly related to the energy and absorbed doses of radiation applied during irradiation. These free radicals can significantly affect the mechanical properties of the material [46].

However, the subsequent reactions of free radicals depend on several factors, such as the chemical structure of the polymer, the presence of oxygen, and the irradiation conditions. Once formed, these radicals can undergo recombination, leading to crosslinking, or they may react with surrounding molecules, initiating oxidative degradation or chain scission [47].

### 2.2. Irradiation Conditions

When polymers are irradiated, the interaction begins at the molecular level. These interactions lead to the formation of reactive intermediates, such as free radicals, which play a key role in determining the results of the irradiation process [48]. It is possible to influence these reactions and achieve desirable effects, such as crosslinking, grafting, or degradation, by controlling factors such as

- Chemical structure;
- Temperature of irradiation;
- Radiation type;
- Radiation absorbed doses;
- Atmosphere of irradiation.

But, the main effect will depend on the polymer chain [47,49].

#### 2.2.1. Influence of Chemical Structure

The chemical structure of the polymer plays a crucial role in its response to ionizing radiation as the molecular arrangement and the nature of the chemical bonds determine whether the material will undergo crosslinking, degradation, or other effects. In polymers composed of strong covalent bonds, such as polyethylene and polypropylene, irradiation can induce crosslinking, increasing the material’s thermal and mechanical resistance.

On the other hand, polymer degradation occurs when ionizing radiation breaks the main chain, reducing molecular weight and altering its physicochemical properties. Susceptibility to degradation is not related to the presence of weak bonds but rather to the polymer’s chemical structure. In the case of polymers containing aromatic groups, such as polystyrene, the electronic stabilization provided by the resonance of the benzene ring can help to dissipate some of the received energy, making these materials more resistant to radiation-induced degradation. However, resistance depends on the absorbed dose and the irradiation environment conditions [50,51,52,53,54].

An example of how chemical structure influences the effects of radiation can be observed when comparing PP and PS. PS is more resistant to ionizing radiation than PP due to its aromatic rings, which stabilize the molecule through electronic resonance. This stabilization helps to dissipate the energy absorbed during irradiation, reducing chain scission and oxidative degradation. In contrast, PP is more susceptible to radiation-induced degradation as it predominantly undergoes chain scission, leading to reductions in molecular weight and mechanical strength. Additionally, the presence of methyl groups in PP facilitates oxidative reactions under irradiation, further accelerating degradation. As a result, while PS can maintain its integrity under radiation exposure, PP often requires stabilizers to prevent excessive degradation [10,55,56,57].

#### 2.2.2. Influence of Temperature on Polymers

There is a significant difference in the degradation caused by the irradiation of polymers in the solid state compared to the molten state. To achieve this phase change, heat must be added to the material [49,58].

The increase in temperature might cause the formation of vacancies in crystalline material, thereby enhancing diffusion within it. In partially crystalline polymers, these vacancies expand the free volume, facilitating the diffusion of substances such as oxygen, reagents, or free radicals [59,60].

The diffusion process occurs through a series of jumps, where the free volume is created by the movement of polymer segments into spaces adjacent to the moving molecule. Above the glass transition temperature ($T_{g}$), the mobility of small molecules in polymers indicates that chemical reactions can occur almost as rapidly as in a solution. Additionally, it is important to note that, above $T_{g}$, polymers show greater molecular flexibility, which favors relaxation processes and structural rearrangements. These phenomena enhance the interaction between reactive species and the polymeric matrix, facilitating chemical or physical modifications induced by irradiation [49,61].

#### 2.2.3. Influence of Radiation Type

**Figure 3:**
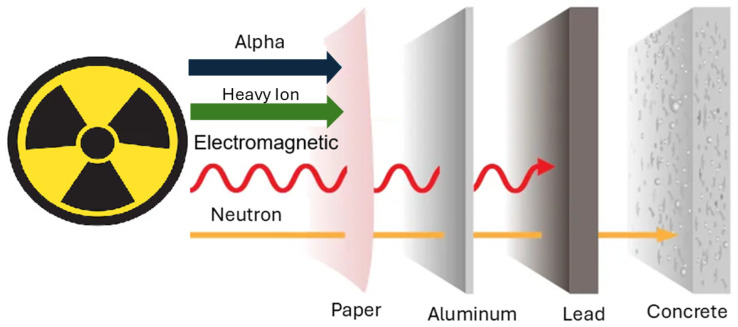
Penetration of ionizing radiation.

The penetration depth of alpha particles and heavy ions is significantly lower compared to gamma radiation and neutrons. Consequently, when a low-density polymer is irradiated with gamma radiation, it can be assumed that the material is uniformly irradiated throughout its entire volume [62,63,64,65]. Figure 3 illustrates the penetration capabilities of alpha particles, heavy ions, electromagnetic radiation, and neutrons.

**Figure 4:**
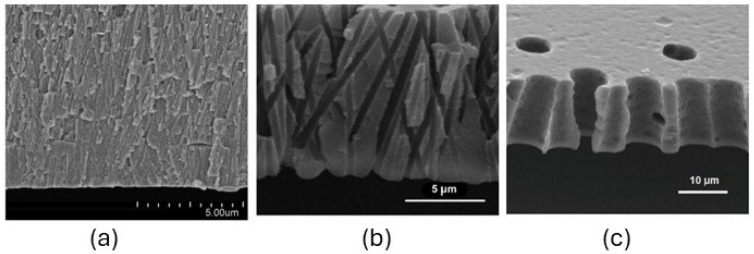
Cross-sections of typical track-etched membranes with nominal pore sizes of (**a**) 0.03, (**b**) 0.8, and (**c**) 8 µm [66]. Copyright 2025, Elsevier B.V.

The effects caused by alpha and heavy ion particles are restricted to the material surface due to their limited penetration depth. Additionally, depending on their energy, they can induce localized damage and, in some cases, even create small perforations in thin polymer films [66], as shown in Figure 4.

**Figure 5:**
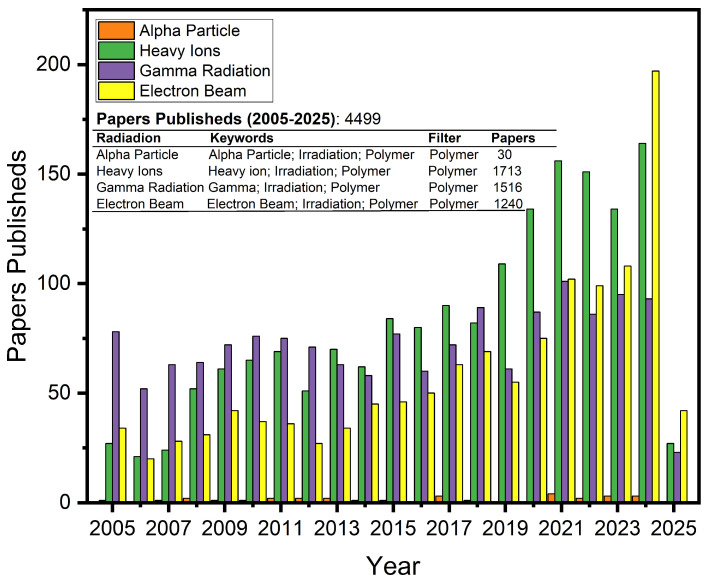
Papers published by type of radiation.

For comparison purposes, a survey was conducted on the SCOPUS platform of papers published between January 2005 and 20 April 2025. Figure 5 shows how this search was conducted. As can be seen, the topics with the highest number of publications are heavy ions and gamma radiation.

**Figure 6:**
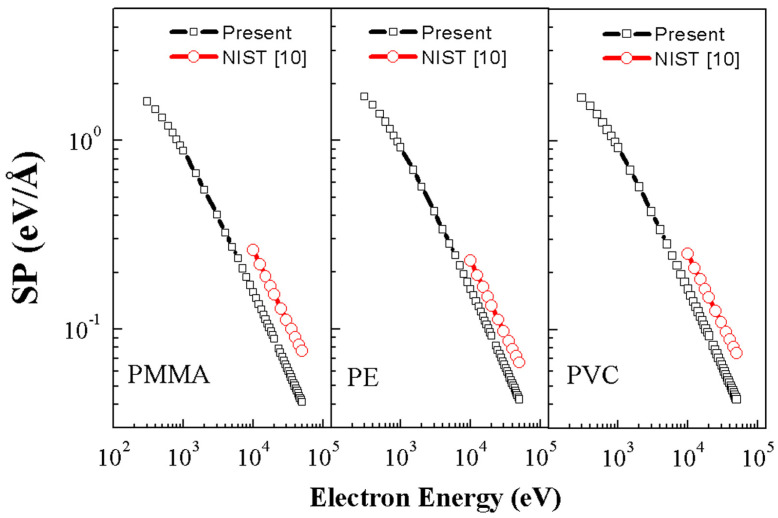
Comparison of the stopping power calculated with NIST. Reprinted with permission from Ref. [65]. Copyright 2015, Elsevier B.V. Licensed under CC BY 4.0.

As illustrated in Figure 6, the higher the incident electron energy in the materials, the greater the energy deposition and penetration into polymers such as poly(methyl methacrylate) (PMMA), polyethylene (PE), and Polyvinyl Chloride (PVC). This effect is particularly relevant in radiation processing, where precise control of electron energy allows for modifications at different depths, enhancing material properties or inducing structural changes [65].

#### 2.2.4. Influence of the Absorbed Dose

The influence of the absorbed dose on polymers is a crucial aspect that affects their physical, chemical, and mechanical properties. The absorbed dose (Gy) represents the amount of energy absorbed per unit mass of the irradiated material [30,67].

Exposure to radiation can induce chemical reactions in polymers, leading to the formation of functional groups, such as crosslinking, grafting, and degradation of the polymer structure. This can result in changes in chemical properties, such as chemical stability and solvent resistance [6,68,69,70].

High absorved doses of radiation can cause significant molecular degradation in polymers, leading to the breaking of molecular chains and a reduction in the mechanical properties. This can result in the loss of strength, ductility, and toughness of the material [71,72].

#### 2.2.5. Influence of Atmosphere on Irradiation

The atmosphere present during irradiation significantly influences the outcome of the process by facilitating the incorporation of smaller molecules or particles into the polymer matrix [49,73].

**Figure 7:**
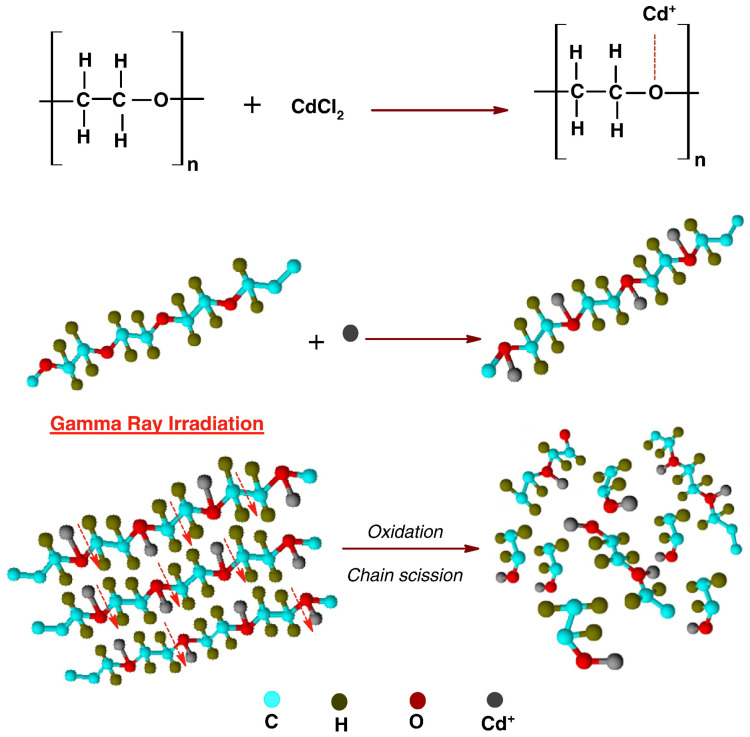
Polymer structure and chain oxidation during irradiation. Reprinted with permission from ref. [75]. Copyright 2015, Elsevier B.V.

One of the primary challenges associated with irradiation is oxidation, which can cause undesirable changes in the properties of the irradiated polymer [74]. Oxidation in irradiated polymers primarily occurs due to the interaction between oxygen and free radicals generated during exposure to ionizing radiation. Oxygen, because of its high reactivity and electronegative nature, readily reacts with these radicals. In oxygen-rich environments, the free radicals within the polymer rapidly interact with oxygen molecules, forming oxidized species. Figure 7 illustrates the process, showing the polymer chain of polyethylene oxide–cadmium chloride PEO–CdCl_2_ that bonds to oxygen molecules [75].

**Figure 8:**
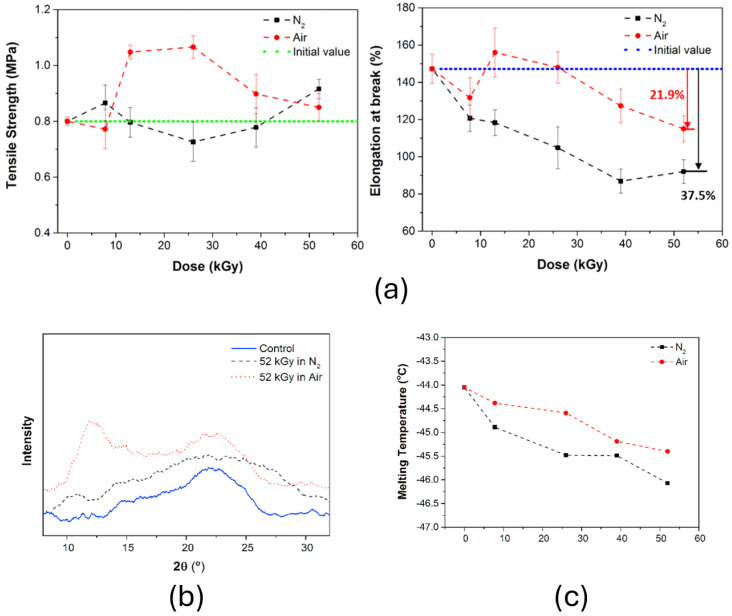
(**a**) Tensile strength and elongation at break; (**b**) XRD patterns of samples before and after radiation; (**c**) changes in *_T_m* on DSC curves of samples irradiated with different absorbed doses. Reprinted with permission from ref. [76]. Copyright 2016, Elsevier B.V.

In order to show that the irradiation atmosphere causes significant changes in the results, Liu et al. [76] investigated the combined effects of fast neutron and gamma radiation on the mechanical, thermal, and chemical behavior of silicone foams under air and nitrogen atmospheres. highlighting significant changes due to the synergistic interaction between the two types of radiation. Mechanically, tensile strength initially increased and then decreased at higher absorbed doses, while elongation at break consistently decreased, with a more pronounced reduction in nitrogen (37.5%) compared to air (21.9%), as shown in Figure 8a, attributed to increased crosslinking density. Structural analysis using two-dimensional XRD revealed short-range ordered regions in foams irradiated in air, while nitrogen promoted a more amorphous state, as illustrated in Figure 8b. Thermal analysis indicated a decrease in the crystalline melting temperature with increasing radiation absorbed dose, confirming reduced crystallinity due to a higher crosslinking density, as demonstrated by the DSC curves in Figure 8c.

## 3. Applications of Irradiated Thermoplastic Polymers

Polymer irradiation with different types of radiation, such as gamma rays, X-rays, electron beams, alpha particles, and neutrons, plays a key role in the development of advanced materials with enhanced properties for a wide range of applications. This process can structurally modify polymers, promoting effects such as crosslinking, controlled degradation, and grafting of new molecular components. These changes result in materials with superior mechanical, thermal, optical, and dielectric characteristics, meeting the demands of various industries, including nuclear, electronics, robotics, and additive manufacturing [77,78,79,80,81,82,83].

In light of these limitations, the irradiation of thermoplastic polymers emerges as a promising alternative to enhance their properties and broaden their applications. The following subsections will explore the state of the art on this topic, highlighting the most recent advancements in the use of irradiated polymers across various fields.

### 3.1. Classification Based on Polymer Type

Understanding the effects of irradiation on thermoplastic polymers enables a deeper understanding of their physical, chemical, mechanical, and microstructural behavior, allowing their application in desired uses. Table 1 provides an overview of some well-known polymers in the literature, highlighting the commonly applied absorbed doses, the resulting effects after irradiation, and the main characteristics of these irradiated polymers.

Table 1 presents a summary of the main polymers, including the typically absorbed doses and the resulting effects. In the following subsections, the characteristics of some of the most relevant polymers and the changes they undergo after irradiation will be discussed.

#### 3.1.1. Polyethylene

Polyethylene (PE) is a widely used polyolefin, notable in various formulations as an insulating material for low- and medium-voltage cables in nuclear power plants, where radiation is part of the material’s normal environment. Consequently, many studies focus on PE performance, particularly in its crosslinked form (XLPE), evaluating different levels of degradation (e.g., for varying absorbed doses at different dose rates). In the short term, PE can tolerate absorbed doses on the order of 10 kGy (1 Mrad) or more, depending on the thermal environment, its crystalline structure (amorphous, semicrystalline, or highly crystalline), the degree of crosslinking, and the specific requirements of each application [84]. Radiation exposure can promote both crosslinking and chain scission; at moderate levels, crosslinking tends to improve thermal stability and certain mechanical properties, while very high absorbed doses can lead to material degradation. Nonetheless, at room temperature, PE still exhibits satisfactory mechanical performance, including good tensile elongation and tensile strength, even after radiation exposure [85].

Svoboda et al. [86] investigated the influence of irradiation on the crystallization of high-density polyethylene (HDPE) at 200 °C, with absorbed doses ranging from 0 to 120 kGy, and observed improvements in properties at 30 and 60 kGy, whereas higher absorbed doses resulted in polymer degradation. Sirin et al. [87] analyzed the influence of irradiation on the mechanical properties of low-density polyethylene (LDPE) blends with polypropylene (PP), using absorbed doses of up to 100 kGy, and verified that degradation occurred due to excessive increases in the absorbed dose.

#### 3.1.2. Polypropylene

Polypropylene (PP) is a radiation-degradable polymer. Unlike polyethylene (PE), PP undergoes chain scission when exposed to ionizing radiation, leading to a reduction in molecular weight and mechanical strength. The presence of methyl groups in the main chain influences its radiation response, making it less tolerant to high doses compared to PE. Pomerantz et al. [88] did not specifically mention isolated PP but rather discussed ethylene–propylene rubber (EPR), which is known to have greater radiation resistance.

A critical application of PP is in the medical industry, where gamma radiation is used for sterilization. However, in these cases, radiation stabilizers are employed to prevent PP from degrading under radiation exposure and losing its mechanical properties. Without proper stabilization, PP experiences oxidative degradation, which reduces its molecular weight and crystallinity. Liu et al. [89] investigated the influence of gamma irradiation up to 1000 kGy on different types of polypropylene and observed that higher doses lead to lower melting points, decreased crystallinity, and reduced molecular weight due to increased oxygen uptake, which accelerates the aging degradation of the polymer chain. Additionally, while PP itself is susceptible to degradation, when incorporated into elastomers, its mechanical resistance can be enhanced [90,91,92].

Considering these factors, it is important to clarify that PP does not exhibit the same high tolerance to radiation as PE. Instead, it requires stabilization strategies to withstand radiation exposure, particularly in applications where long-term mechanical integrity is required.

#### 3.1.3. Polyamide

Polyamides, such as nylon, Kevlar 29, and others, can be synthesized in various ways, enabling applications ranging from textiles (like fibers and woven yarns) to rigid plastics (such as Nylatron^®^ and Nycast^®^, among others) [93]. From a thermo-oxidative standpoint, several studies examine the aging of nylon, indicating that it is a relatively robust material [94,95] However, there is still limited knowledge about the mechanical behavior of polyamides when exposed to radiation environments.

Birkinshtaw et al. [96] investigated the effects of gamma radiation on Nylon 6.6 for medical applications under high absorbed doses. Their results showed that exposures of up to approximately 25 kGy (2.5 Mrad) led to about a 5% reduction in the material’s mechanical properties. This behavior is similar to that observed in polyolefins, such as PE and PP. Further studies on the effects of radiation on polyamides can be found in the literature [97,98].

Although discussions on the effects of radiation generally focus on polyolefins, research indicates that, due to the structural organization of the functional groups that comprise the polymer backbone, the overall resistance to radiation can increase. Thus, radiation-induced degradation sensitivity can be considered a relevant factor for aliphatic polyamides, such as nylon.

#### 3.1.4. Acrylonitrile–Butadiene–Styrene

Acrylonitrile–butadiene–styrene (ABS) is a widely used copolymer due to its ease of processing, lightweight nature, mechanical and chemical resistance, and good thermal stability [99]. This material is commonly used in various industrial applications, including automotive components, electronic devices, and exterior coatings. Its molecular structure consists of three monomers: acrylonitrile, which provides chemical resistance and thermal stability; butadiene, responsible for toughness and impact resistance; and styrene, which imparts rigidity and processability [100,101].

The effects of ionizing radiation on ABS have been widely studied, particularly in the context of material degradation and aging. Although most research focuses on exposure to UV light and photo-oxidation, gamma radiation can also significantly impact its mechanical and structural properties. Exposure to high absorbed doses can promote crosslinking reactions and degradation to varying degrees, depending on the absorbed dose and environmental conditions [102].

In moderate absorbed doses, ABS can retain its mechanical properties without major alterations [103], making it suitable for applications where radiation exposure is limited. However, higher doses tend to induce crosslink formation and degradation of the elastomeric phase of butadiene, reducing deformation capacity and making the material more brittle. This phenomenon occurs due to polymer chain fragmentation and microstructural changes, leading to decreased elongation and modification of overall mechanical characteristics [104].

Additionally, the absorbed dose rate directly influences the predominant degradation mechanisms. Under non-oxidative conditions, crosslinking may be the dominant effect, resulting in increased thermal and chemical resistance of the material. In contrast, exposure to oxygen during irradiation can accelerate oxidative degradation, promoting polymer chain scission and loss of structural integrity [105,106].

#### 3.1.5. Polycarbonate

Polycarbonate (PC) is a thermoplastic widely used in electronics—such as in resistors, capacitors, and data storage devices—owing to its excellent heat resistance and flame-retardant properties [107]. As a result, its behavior under radiation has been the subject of extensive study. In one investigation, the mechanical properties of PC were evaluated under electron beam irradiation at absorbed doses up to 250 kGy (25 Mrad) with a beam energy of 2 MeV. Notably, this study revealed that PC maintained its tensile elongation and strength for absorbed doses below 50 and 100 kGy, respectively, even when compared to polymers typically irradiated using 60Co sources (which emit discrete energies of 1.17 and 1.33 MeV) [108].

A similar analysis focused on a Lexan Margard variant of polycarbonate under gamma irradiation using a 137Cs source (with an average energy of approximately 1.176 MeV) operating at a dose rate of 2 kGy/h. This work found that the elongation at break was the most sensitive property, showing a decline even at low absorbed dose, while tensile strength and fracture toughness remained relatively stable for absorbed doses under approximately 20 kGy (2 Mrad). These findings confirm that PC is inherently robust against low to moderate absorbed doses, a characteristic that supports its reliability in applications where exposure to ionizing radiation is a concern [109].

Additionally, the observed resistance to degradation may be attributed to the polymer’s stable molecular structure, which can benefit from limited crosslinking processes induced by radiation.

### 3.2. Application of Irradiated Thermoplastic Polymers

In Table 2, the main applications of irradiated polymers are highlighted, showcasing how the unique properties of these materials can be exploited to address specific technological challenges. The corresponding references reinforce the scientific and practical relevance of these applications.

Considering the application of irradiated polymers in the field of additive manufacturing, we can take the work by Wady et al. [110] as an example. In that study, the authors investigated the effects of gamma irradiation on polymers produced via additive manufacturing using the fused filament fabrication (FFF) process. Commercial filaments of PLA, TPU, CPE, nylon, ABS, and PC were exposed to rabsorbed doses of up to 5.3 MGy, allowing for the evaluation of the evolution of these materials’ mechanical and structural properties. The results indicated that the behavior of polymers under irradiation depends strongly on both their chemical composition and the microstructure inherent to the printing process. In particular, nylon demonstrated superior performance by maintaining virtually unchanged tensile strength and exhibiting an increase in elastic modulus with absorbed dose, although the increase in adhesiveness at very high absorbed doses may limit its use in moving parts. In contrast, PLA showed high radiation sensitivity, with approximately a 50% reduction in tensile strength and elastic modulus at significantly lower absorbed doses, demonstrating the predominance of chain scission mechanisms in this polymer. The authors also observed that irradiation induces concurrent processes, such as the formation of crosslinks and the cleavage of polymer chains, which are modulated by the porosity and anisotropy characteristic of 3D printed components. The influence of oxygen diffusion, related to the internal structure of the filaments, proved to be a key factor in the radiolytic degradation mechanisms. By selecting appropriate materials and fine-tuning printing parameters, this work advanced the state of the art by demonstrating that it is possible to produce 3D components that maintain structural performance in high-radiation environments, thereby expanding the range of applications for polymers produced via additive manufacturing.

Moreover, considering the advanced manufacturing sector, for the application of additive manufacturing, West et al. [113], using Polylactic Acid (PLA), demonstrated that γ irradiation significantly modifies its properties when 3D-printed. The elastic modulus, tensile strength, and hardness increased with absorbed doses, while ductility and toughness decreased. Chemical analyses revealed that gamma irradiation induces crosslinking in PLA, forming crosslinks between the polymer chains.

**Figure 9:**
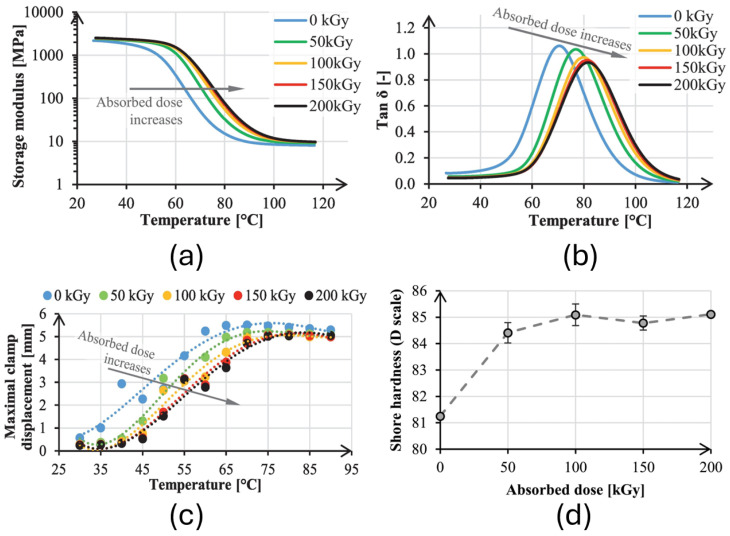
(**a**) Different storage moduli; (**b**) loss factor (tan(δ)); (**c**) maximal clamp displacements of the bending tests of specimens irradiated by different absorbed doses; (**d**) Shore D hardness of VeroWhite specimens with different irradiation regarding absorbed doses [200]. Copyright 2025, Elsevier B.V. Licensed under CC BY 4.0.

In the application of additive manufacturing, Krizsma et al. [200] investigated a methodology for improving molds for polymer jetting. The study revealed that irradiation significantly improved the stiffness, dimensional stability, and thermal resistance of the molds, optimizing their industrial applicability. The main results include a 10 °C increase in the glass transition temperature (*T_g_*), which rose from 70.8 °C in non-irradiated molds to 81.6 °C in molds irradiated at 200 kGy, as demonstrated in Figure 9a,b. Additionally, the storage modulus indicated greater stiffness, with saturation observed at absorbed doses of 150–200 kGy, as shown in Figure 9c, while the Shore D hardness increased from 81.3 to 85.1, as presented in Figure 9d.

**Figure 10:**
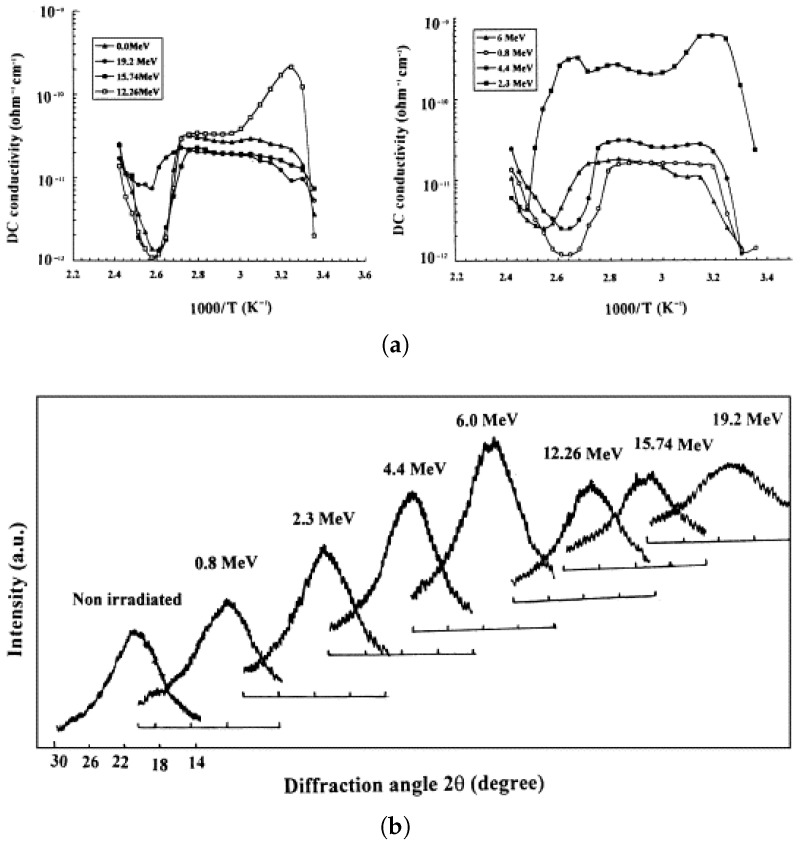
(**a**) Comparison of electrical conductivity of irradiated and non-irradiated polymers with neutrons; (**b**) XRD in the 14 to 30° range of irradiated and non-irradiated CR-39. Reprinted with permission from ref. [201]. Copyright 2003, Elsevier B.V.

Considering the diverse applications of irradiated polymers in the nuclear industry, one example addressing polymer irradiation is the work by Nouh et al. [201], who investigated the effect of fast neutron irradiation in the range of 0.8 to 19.2 MeV on the electrical, optical, and structural properties of polyallyldiglycol carbonate (CR-39) for use in solid-state nuclear track detectors. They observed that the fast neutron flux caused degradation in the material, resulting in changes in its electrical properties, as illustrated in Figure 10a, which shows the electrical conductivity as a function of temperature. The results of the X-ray diffraction test, performed between 14° and 30°, are presented in Figure 10b. The XRD spectra indicated that neutron irradiation at energies between 6 and 19.2 MeV caused a reduction in the crystallinity of the material.

**Figure 11:**
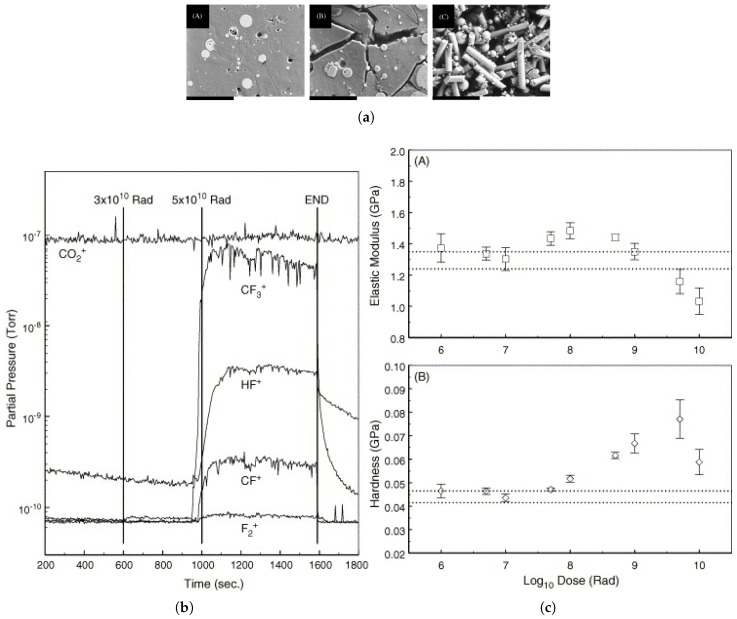
(**a**) SEM for samples with absorbed doses (subfigures (A) 10^9^, (B) 10^10^, and (C) 10^11^ rad); (**b**) residual gas analysis during α particle irradiation of PTFE; (**c**) elastic modulus of PTFE irradiated with α particles, Subfigure (A), and hardness of PTFE irradiated with α particles, Subfigure (B). Reprinted with permission from ref. [202]. Copyright 2006, Elsevier B.V.

Another study focused on the application of irradiated polymers in the nuclear field is that of Fisher et al. [202], in which the authors irradiated a 2 mm thick glass fiber-reinforced PTFE plate with α particles generated by the radionuclide Pu-238, with a characteristic energy of 5.5 MeV. This study was conducted with a potential application within nuclear reactors in mind. The primary failure mechanisms observed in the PTFE matrix irradiated with alpha particles at high absorbed doses are chemical degradation and mass loss at the surface, as illustrated in Figure 11a. The radiolytic decomposition of the polymer matrix results in the release of volatile molecules from the surface, as revealed by the residual gas analyzer shown in Figure 11b. It was concluded that α particle irradiation in PTFE mainly results in crosslinking at absorbed doses below $10^{10}$ rad (approximately 100 MGy), accompanied by lower levels of fragmentation, branching, and unsaturation. In fact, the highest level of crosslinking occurs at approximately 50 MGy, where a peak in hardness is observed, as demonstrated in Figure 11c.

**Figure 12:**
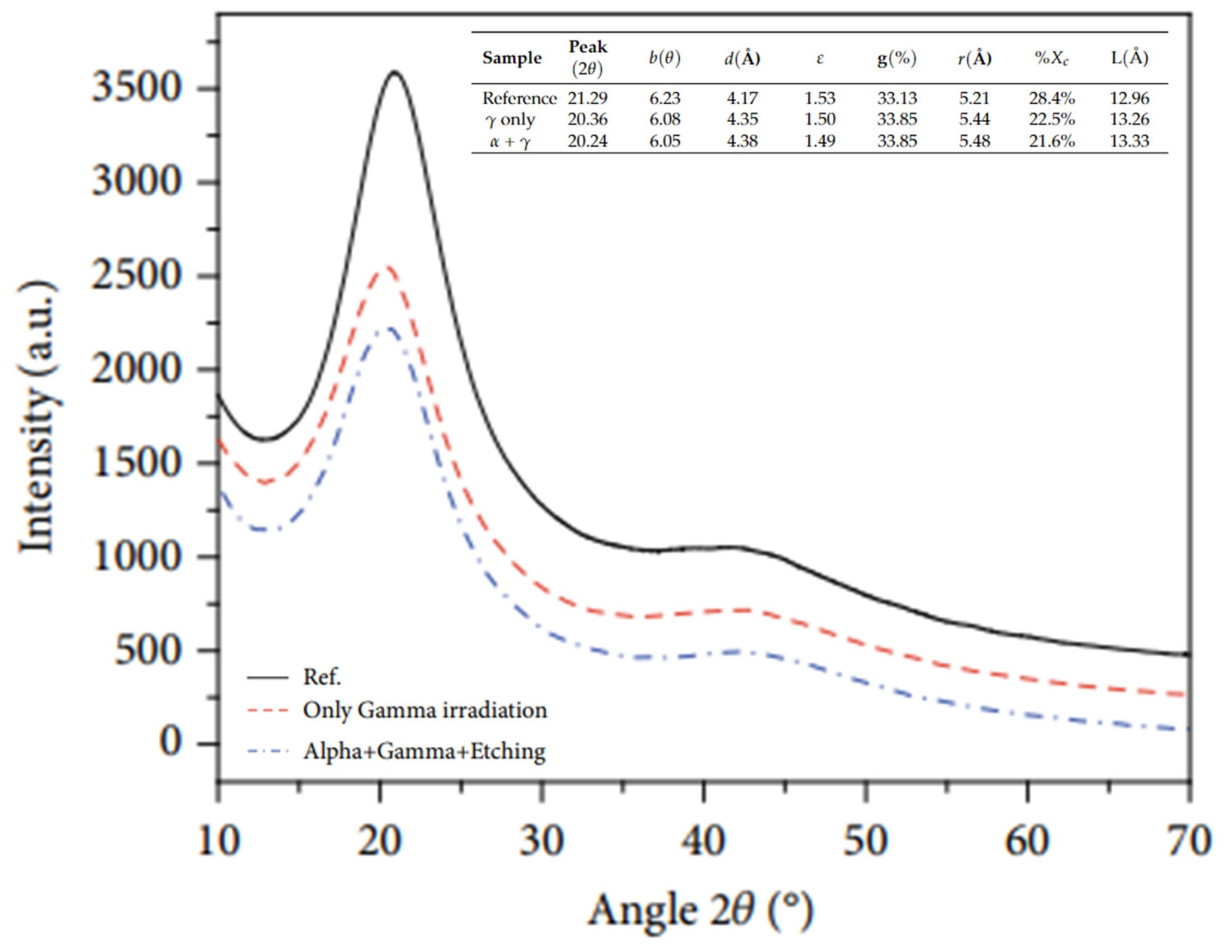
Comparison of the XRD spectra of PM-355 irradiated in the range of 10 to 70°. Reprinted with permission from ref. [203]. Copyright 2021, Wiley. Licensed under CC BY 4.0.

Rahaman et al. [203] conducted a study on the effect of γ and alpha radiation on the crystallization of PM-355. For the experiment, a sheet with a thickness of 500 µm and a density of 1.32 gcm^−3^ was used. Gamma irradiation was performed with a Co-60 source and alpha irradiation was performed with an Am-241 source. Based on the analysis performed on the diffractogram shown in Figure 12, it can be concluded that the interplanar distance, the polymer chain distance, distortion, and crystal size increased, while the microstrain and crystallinity index decreased after irradiation. These changes in parameters are likely due to internal stresses generated after irradiation and chemical attack.

**Figure 13:**
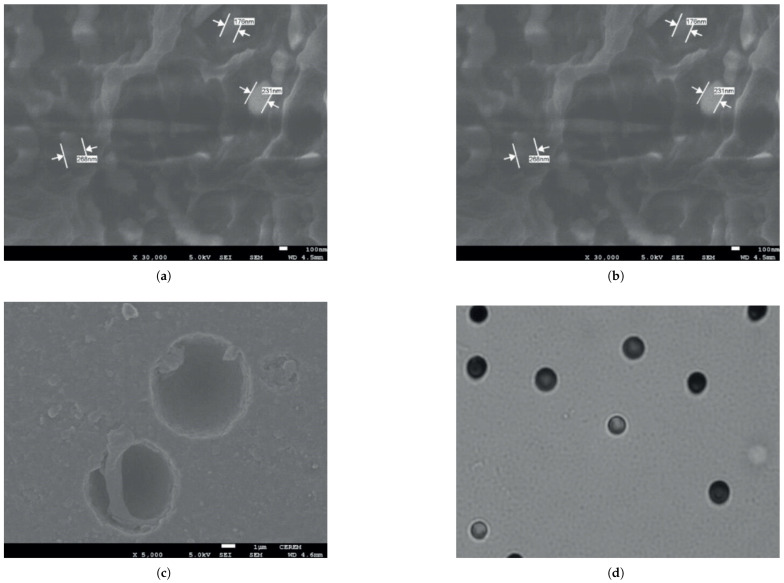
(**a**) SEM reference image; (**b**) gamma radiation only; (**c**) gamma and alpha radiation; (**d**) track caused by alpha particle. Reprinted with permission from ref. [203]. Copyright 2021, Wiley. Licensed under CC BY 4.0.

Figure 13a–d show images obtained by electron scanning microscopy (SEM) of PM-355, irradiated with gamma radiation only and irradiated with alpha and gamma radiation followed by chemical attack, respectively. Figure 13a reveals that the grains of the reference sample have an almost spherical shape, with a diameter in the range of 100–170 nm. After gamma irradiation, Figure 13b shows an increase in the size of these grains. The surface of the gamma-irradiated sample also becomes rougher compared to the reference. This SEM result from electron microscopy is consistent with the XRD analysis, indicating that the polymer crystal size increased after gamma irradiation. However, it is important to note the difference between the crystal size and grain size.

The grain size is much larger than the crystal size, indicating that the grain is formed by the accumulation of several crystallites. Figure 13c,d show images captured, respectively, by SEM and by a common optical microscope, of samples irradiated with alpha and gamma radiation followed by chemical attack. These images reveal circular nuclear tracks caused µm [203].

In the biomedical field, irradiation, whether ionizing or non-ionizing, has a range of applications, from sterilizing instruments to irradiating implants and polymers used in drug delivery. A study that effectively describes irradiation in biomedical applications is the research by Roxby et al. [204], which investigates the effects of gamma and X-ray irradiation on polymers widely used in the biomedical industry. The analyzed polymers include high-density polyethylene (HDPE), low-density polyethylene (LDPE), and polypropylene (PP), with four variants of each type. The primary objective was to evaluate modifications in the physicochemical properties of the materials to determine the equivalence between irradiation methods, considering parameters such as absorbed doses and dose rate. The materials were exposed to absorbed doses of 30 kGy and 55 kGy, reflecting the minimum and maximum levels used in industrial sterilization. The dose rate was standardized for each technology: an average of 34 kGy/h for X-rays and 11 kGy/h for gamma radiation. Additionally, some tests evaluated different dose rates (1 to 80 kGy/h) to verify potential effects related to irradiation speed. Material characterization was performed using differential scanning calorimetry (DSC), attenuated total reflectance infrared spectroscopy (ATR-FTIR), and gel permeation chromatography (GPC), allowing for the evaluation of changes in melting temperature (Tm), chemical structure, and molecular weight distribution. For HDPE, the DSC results indicated no significant change in Tm between the gamma- and X-ray-irradiated samples. ATR-FTIR spectroscopy did not reveal the appearance or disappearance of spectral peaks, indicating chemical stability, while the GPC analysis showed a slight trend toward increased molecular weight due to crosslinking, with no significant differences between the irradiation types. For LDPE, the Tm values remained within the statistical equivalence margin, with no significant changes in the infrared spectra. The GPC analysis indicated a trend similar to that of HDPE, with more pronounced crosslinking formation at higher absorbed doses. In the case of PP, a slight reduction in Tm was observed with increasing absorbed doses but without relevant differences between gamma and X-rays. ATR-FTIR spectroscopy did not indicate any significant chemical alterations, while the GPC analysis revealed a trend toward chain scission, reducing the molecular weight of irradiated PP, regardless of the technology used. The results indicate that the changes induced by gamma and X-ray irradiation are equivalent under the tested conditions. None of the materials exhibited significant differences in chemical structure or thermal properties. The trend toward crosslinking in PE and chain scission in PP was similar between the radiation types, suggesting that both techniques are suitable for sterilizing medical devices containing these polymers. The research demonstrated that replacing gamma irradiation with X-rays is feasible for the industrial sterilization of HDPE, LDPE, and PP while maintaining material integrity.

In the field of environment and energy storage, polymer irradiation enables the enhancement of properties for applications such as photovoltaic cells, leading to more efficient solar energy storage systems. In the context of environmental technologies and energy storage, the irradiation of polymers has proven to be an effective strategy for enhancing material properties, particularly in applications such as photovoltaic cells. A study conducted by Sebak et al. [205] focused on improving the efficiency of polymer solar cells (PSC) based on composites composed of chitosan (CS), polyvinyl alcohol (PVA), and reduced graphene oxide (rGO) treated with gamma radiation. The rGO nanoparticles were exposed to gamma rays at doses of 5, 10, 15, 20, and 25 kGy, with the aim of modifying their structural and chemical characteristics. The results demonstrated that gamma irradiation played a crucial role in reducing structural defects in rGO and in promoting the formation of oxygen-containing functional groups. These modifications significantly improved the compatibility of rGO with the polymeric matrix, resulting in composites with enhanced electrical conductivity, increased thermal stability, and more homogeneous phase distributions. When applied in photovoltaic devices, these composites led to a notable increase in solar cell efficiency. Specifically, the efficiency of devices incorporating non-irradiated rGO was approximately 1.1%, while those using rGO irradiated at 25 kGy reached around 2.2%, indicating a performance improvement of about 100%.

Additionally, gamma irradiation contributed to the microstructural refinement of the composite layers, enhancing their uniformity and enabling the formation of more efficient interfaces for charge transport. This structural reorganization was essential for minimizing crystalline defects that typically act as traps for charge carriers. Improvements in conductivity, associated with the reduction in charge-transfer resistance (Rct), suggest increased mobility of electrons and holes—an essential factor for high-performance photovoltaic operation. The reduction in charge recombination, facilitated by the structural effects of irradiation, resulted in higher short-circuit current densities and improved overall energy conversion efficiency. Among the tested doses, the treatment at 30 kGy proved to be the most effective, offering the optimal balance between microstructural enhancement and photovoltaic performance. Lower doses were insufficient to promote significant improvements, while excessively high doses could compromise the structural integrity of the material [205].

Gamma irradiation, by modifying the microstructure, promoted increased uniformity and homogeneity of the layers, contributing to the formation of more efficient interfaces for charge transport. This structural modification was crucial for minimizing crystalline defects, which typically act as charge carrier traps, reducing solar cell efficiency. The increased conductivity, attributed to the reduction in charge-transfer resistance (Rct), suggests improved mobility of electrons and holes, which is essential for cell performance. The reduction in charge recombination, facilitated by irradiation, resulted in higher short-circuit current densities and significantly improved energy conversion efficiency, particularly at 30 kGy absorbed doses. These findings underscore the importance of precise adjustments to the absorbed doses as lower absorbed doses were not sufficient to promote significant changes, while excessively high absorbed doses could potentially compromise material stability. In this study, the 30 kGy absorbed doses were ideal, providing the best balance between structural improvement and photovoltaic efficiency.

**Figure 14:**
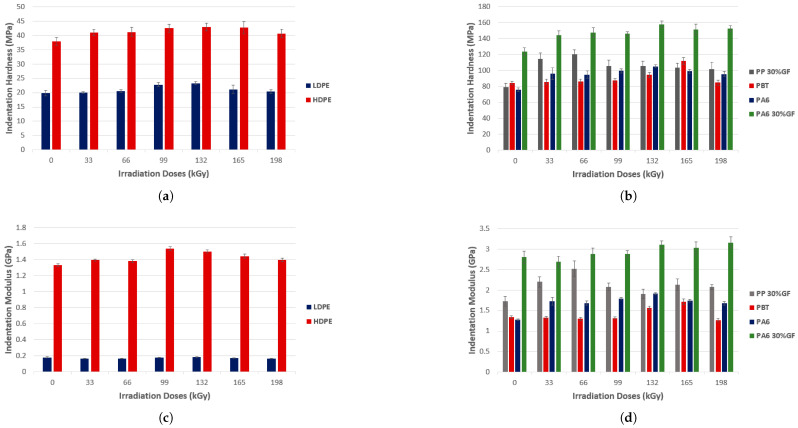
(**a**) Indentation hardness; (**b**) indentation hardness; (**c**) indentation modulus ($E_{IT}$); (**d**) indentation modulus. Reprinted with permission from ref. [206]. Copyright 2018, MDPI AG. Licensed under CC BY 4.0.

Considering the application of irradiated polymers for consumer goods, we can improve the barrier, thermal, and mechanical properties of polymers for use, for example, in food packaging. Based on this principle, David et al. [206] investigated the effects of electron beam radiation on the micromechanical and thermomechanical properties of various polymers, including low-density polyethylene (LDPE), high-density polyethylene (HDPE), polypropylene (PP) with glass fibers, poly (butylene terephthalate) (PBT), Nylon 6 (PA6), and poly (nonamethylene terephthalamide) (PA9T), revealing significant improvements in their properties due to crosslinking induced by The indentation increased by up to 17% for LDPE and 14% for HDPE at an absorbed dose of 132 kGy, as shown in Figure 14a. Beyond this absorbed dose, hardness decreased as a result of material degradation. For PP reinforced with 30% glass fibers, the hardness increased by 51% at 66 kGy, as shown in Figure 14b. HDPE EIT increased by 16% at 99 kGy, followed by a decrease at higher absorbed doses, as shown in Figure 14c. For reinforced PP, the maximum modulus was observed at 66 kGy, with an increase of 42%, as illustrated in Figure 14d.

**Figure 15:**
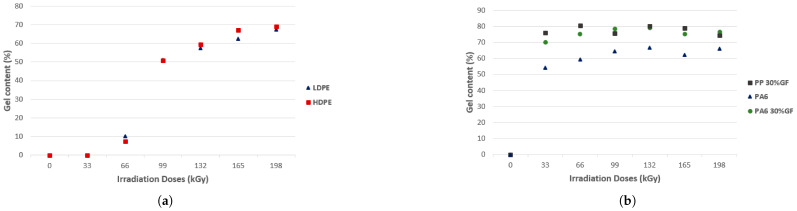

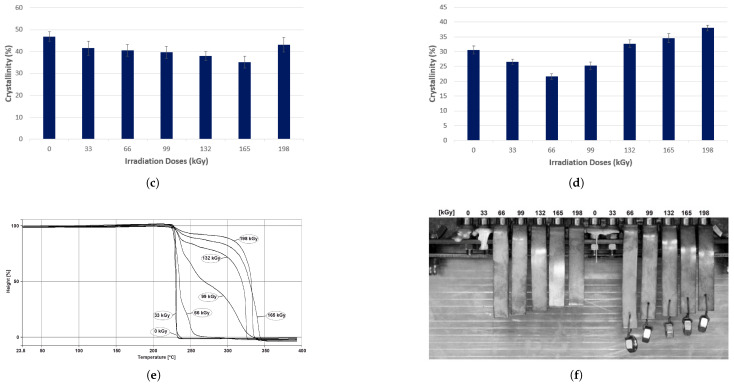
(**a**) Gel content; (**b**) gel content; (**c**) crystallinity (HDPE); (**d**) crystallinity—technical polymers (PP 30% GF); (**e**) TMA(HDPE); (**f**) visual observation after 1 h at 180 °C (HDPE). Reprinted with permission from ref. [206]. Copyright 2018, MDPI AG. Licensed under CC BY 4.0.

Crosslinking, measured as gel content, increased significantly for LDPE and HDPE at 198 kGy, indicating enhanced molecular crosslinking, as demonstrated in Figure 15a. For PP with glass fibers and PA6, the maximum gel content was achieved at 132 kGy, as shown in Figure 15b. The crystallinity analysis using XRD revealed a reduction in the crystalline phase of HDPE with increasing absorbed doses, with the lowest value at 165 kGy, as illustrated in Figure 15c. For reinforced PP, the lowest crystallinity was observed at 66 kGy, corresponding to the highest values of hardness and modulus, as shown in Figure 15d. TMA demonstrated improved thermal stability for HDPE with increasing absorbed doses, with samples irradiated at 198 kGy exhibiting significant resistance to deformation at elevated temperatures, as shown in Figure 15e. Visual observation confirmed that HDPE irradiated at 198 kGy retained its shape at 180 °C, while non-irradiated HDPE completely melted, as illustrated in Figure 15f [206].

**Figure 16:**
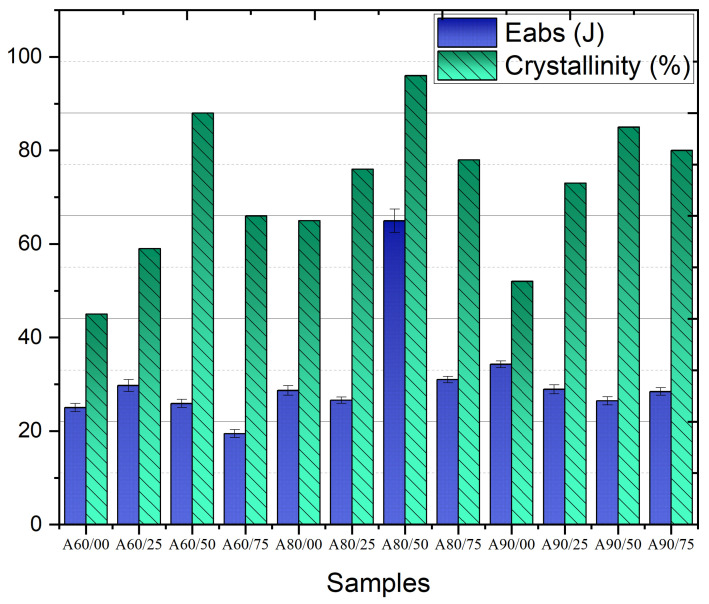
Comparison of absorption energy results of ballistic tests and crystallinity index obtained by XRD in the work of Figueiredo et al. [207]. Copyright 2019, SciELO. Licensed under CC BY 4.0.

For ballistic protection application, Figueiredo et al. [207] investigated the optimal combination of alumina concentration and radiation absorbed dose in Ultra-High-Molecular-Weight Polyethylene (UHMWPE) for ballistic protection applications. The mass proportions of the composite were prepared by mechanical mixing for 10 min and labeled as A00/00, A00/25, A00/50, A00/75, A60/00, A60/25, A60/50, A60/75, A80/00, A80/25, A80/50, A80/75, A90/00, A90/25, A90/50, and A90/75. The first number represents the weight percentage concentration of the ballistic test involved in measuring the residual velocity after the projectile passed through the composite. To analyze the degree of crystallinity of the material, DSC testing was performed, and XRD testing was conducted only for the samples with 80% alumina. The sample that exhibited the highest absorbed energy and crystallinity index was one with 80% alumina and irradiated with an absorbed dose of 50 kGy, as shown in Figure 16.

**Figure 17:**
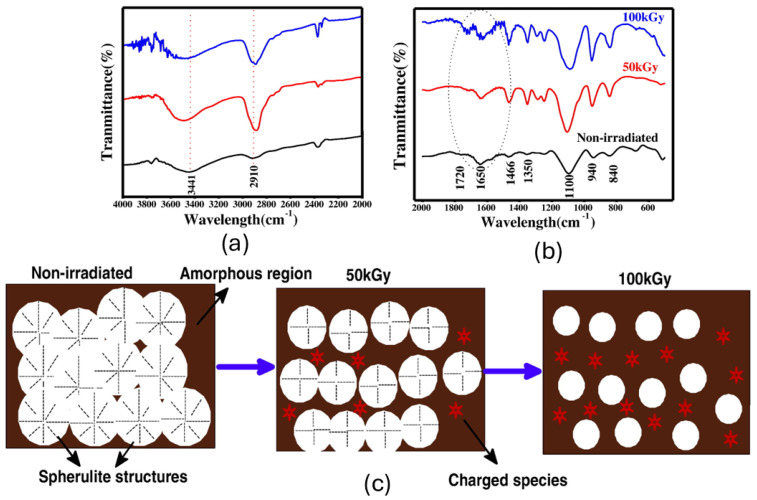
Main results: (**a**) IR spectra between the bands 4000 and 2000 cm^−1^ (Blue: 100 kGy, Red: 50 kGy and Black: Non-Irradiated); (**b**) IR spectra between the bands 2000 and 500 cm^−1^ (1650 cm^−1^ = carbonyl group (C=O); (**c**) representation of spherulitic alterations with absorbed dose. Reprinted with permission from ref. [75]. Copyright 2015, Elsevier B.V.

Considering the molecule grafting section, Raghu et al. [75] investigated the effects of gamma ray irradiation on the physical, chemical, optical, and electrical properties of polymer electrolyte films based on PEO doped with CdCl_2_. FT-IR analysis (Figure 17a,b) revealed degradation through chain scission and crosslinking of polymer chains. Optical microscopy demonstrated changes in morphology, such as reduced spherulitic structures and increased surface roughness, further corroborated by the schematic representation in Figure 17c. The optical properties exhibited increased light absorption, a shift to longer wavelengths, and a reduced optical band gap from 4.25 eV to 3.80 eV, attributed to structural disorder and the formation of carbon clusters. Electrically, the films showed significantly enhanced conductivity, reaching $1.156\times 10^{-4}$, S/cm at 150 kGy due to the increased mobility of the ions from chain scission and the formation of unsaturated bonds. However, the dielectric properties revealed increased permittivity, an electric modulus, and a reduced relaxation time, indicative of non-Debye relaxation behavior.

Batool et al. [208] investigated how grafting Al molecules by ionizing radiation into the PMMA polymer alters the optical property of the composite. The study found that both Al concentration and radiation absorbed dose significantly impacted the absorption coefficient, refractive index, and optical band gap. Increasing the Al concentration and radiation absorbed dose enhanced light absorption and reduced the optical band gap, indicating electronic structural changes. The band gap decreased from 4.21 eV to 3.45 eV in pure PMMA irradiated with 200 Gy, up to 2.95 eV in composites with 15% Al. This reduction was attributed to the formation of defect states and the modification of the electronic structure. The Al-doped composites showed increased refractive index and optical conductivity, enhancing the material’s interaction with light. Gamma radiation also induced structural modifications, including crosslinking and segregation, improving the thermal and mechanical stability of the composites. These characteristics make the materials promising for use in radiation-rich environments, such as the aerospace and nuclear sectors, because of their ability to maintain integrity under prolonged exposure. The combination of Al doping and gamma radiation is thus an effective strategy for optimizing PMMA composites for advanced technological applications.

In the context of ink irradiation, studies on the interaction of UV radiation with inks demonstrate that photopolymerization is influenced by factors such as formulation composition, the presence of photoinitiators and pigments, and the intensity of incident radiation. The curing efficiency depends on the ability of photoinitiators to generate free radicals for the crosslinking of acrylate monomers, with pigments like carbon black potentially hindering this process by absorbing UV radiation, while yellow pigments tend to undergo greater degradation due to their interaction with UV photons. Additionally, UV-induced degradation can occur through direct photodegradation or photoinduced oxidation, leading to the loss of the optical and mechanical properties of the inks. During accelerated aging, the degree of curing may continue to increase in some formulations due to the presence of unreacted reactive species, resulting in the phenomenon of residual curing. The substrate also plays a crucial role as materials more transparent to radiation, such as LDPE, may allow more pronounced ink degradation, whereas substrates less permeable to light, such as PBAT/TPS, may mitigate this effect. In this context, Bardi et al. [209] investigated the behavior of UV-curable inks applied to LDPE and PBAT/TPS substrates, analyzing curing, post-curing, and accelerated degradation. The study evaluated the influence of pigments on UV radiation absorption and their effects on the degree of curing and degradation of the inks. During accelerated aging in a QUV chamber, some samples exhibited residual curing, increasing hardness and gloss, while others suffered discoloration and loss of mechanical resistance. The study also demonstrated that the substrate significantly influences ink degradation, with LDPE allowing more intense degradation compared to PBAT/TPS. These findings reinforce the need to optimize formulations to enhance the durability of inks exposed to UV radiation.

**Figure 18:**
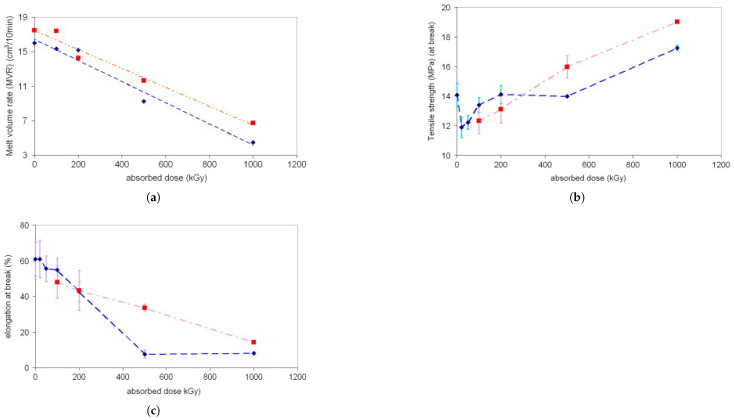
Blue: pre-irradiated PS-HI granulate; Red: irradiated PS-HI injection moulded specimens. (**a**) Melt volume rate; (**b**) tensile strength to fracture; (**c**) elongation (%) of HIPS to fracture. Reprinted with permission from ref. [210]. Copyright 2012, Elsevier B.V.

The field of sustainability, with a focus on recycling, is not a particularly strong area but allows for the reprocessing of thermoplastic polymers through sterilization or complete degradation for disposal. As an example of the use of irradiation in reprocessing, the study by JELčIć and Ranogajec [210] investigated the effect of irradiation on high-impact polystyrene (HIPS), its molding process, and its properties. Figure 18a–c show the results. The early degradation of the pre-irradiated sample, compared to the sample irradiated after processing, highlights the influence of oxygen. Irradiation can lead to polymer oxidation. In the procedure where HIPS granulate is first pre-irradiated and then molded, oxidation is intensified due to the generation of additional radicals per unit of surface area. At very low absorbed doses, oxygen also promotes the oxidative degradation of the main chain of the irradiated polymer. However, at higher absorbed doses and in a nearly inert environment, crosslinking, resulting from the recombination of macroradicals, becomes predominant.

## 4. Final Remarks and Conclusions

This paper presented a review of the effects of ionizing radiation on thermoplastic polymers, highlighting the main concepts of the subject, as well as the scientific advances and practical applications related to this topic. Ionizing radiation, which includes gamma rays, X-rays, and electron beams, has been widely explored since the development of the nuclear industry because of its ability to modify the molecular properties of polymeric materials. These modifications include molecular crosslinking, controlled degradation, molecular grafting, and changes in crystallinity, enabling adaptations to the demands of the industrial, medical, food, and other sectors.

The text highlights the fundamental mechanisms involved in the interaction of radiation with polymers, including ionization, electronic excitation, and the formation of free radicals. These initial processes lead to structural transformations that can be controlled by selecting the type of radiation, absorbed doses, temperature, and environmental conditions. The manipulation of these factors allows for the control and improvement of polymer properties, with better results such as increased thermal and mechanical resistance, chemical functionalization, and biodegradability.

Among the practical applications, the article mentions the use of irradiated polymers in the manufacture of medical devices, food packaging, electrical cables, and aerospace components, as well as examples in ballistic protection. In medicine, these materials offer greater biocompatibility and resistance to radiation sterilization, while, in the food industry, they ensure greater safety and durability of packaged products. In the industrial sector, enhanced resistance to extreme temperature and radiation conditions expands the possibilities for use in high-performance applications.

However, challenges associated with the use of ionizing radiation were also highlighted. Small variations in absorbed doses or environmental conditions can lead to unpredictable results, complicating the standardization of large-scale processes. In addition, some polymers are more sensitive to radiation, limiting their application. To overcome these limitations, recent research explores combinations of radiation with other treatments, such as chemical doping and blending with additives, to improve the stability and functionality of irradiated polymers.

The predictions for future research suggest a focus on optimizing irradiation parameters, such as absorbed doses, temperature, and processing atmosphere, to further expand the application of irradiated polymers. Important characterization techniques, such as FT-IR and XRD, can verify critical properties, such as the degree of crystallinity of the polymer or the formation of specific bands that may arise after crosslinking. These microstructural changes are detected with precision. Furthermore, the development of new polymers, through the formation of blends or composites, represents a promising field, and irradiation may prove to be an important complement in enhancing the properties of these new materials.

In conclusion, the use of ionizing radiation to modify thermoplastic polymers represents a powerful technique with vast application potential. Despite the challenges, scientific and technological advances are pushing the boundaries of what is possible and offer innovative solutions for critical sectors of society. With such research advancements, it is expected that new irradiated materials will emerge that meet the growing demands for performance, sustainability, and innovation.

## Figures and Tables

**Table 1:** Summary of irradiation effects on several polymers, detailing the absorbed doses, observed effects, and relevant characteristics [24].

Polymer	Absorbed Dose	Effects	Relevant Characteristics
PE	50 kGy	Low degradation up to absorbed doses close to 50 kGy; mechanical performance preserved.	High radiation resistance; used in nuclear cables.
PP	40 kGy	Acceptable performance; 40% property loss at absorbed doses above 40 kGy.	High resistance; used in medical devices sterilized by irradiation.
Butyl Rubber	10 kGy	Stress and elongation gradually decrease; hardening at higher absorbed doses (200 kGy).	Used in sealing and wires; maintains basic properties at moderate absorbed doses.
Nylon 6	25 kGy	5% loss in tensile modulus; reduced resistance at higher absorbed doses.	Applications in textiles and rigid plastics.
ABS	50 kGy	Became more brittle (40% reduction in elongation); no resistance loss up to 50 kGy.	Moderate radiation resistance; used in automotive exteriors.
PC	100 kGy	Minimal degradation up to 20 kGy; elongation reduced at higher absorbed doses.	High heat and flame resistance; used in electronics.
RTV-5370	500 kGy	Hardening and increase in storage modulus at high doses (>500 kGy).	Excellent resistance in gasket and cable applications.
PTFE	10 kGy	Significant elongation loss; 40% reduction in tensile strength at high absorbed doses (80 kGy).	Used in chemically resistant environments.
FEP	10 kGy	10% reduction in tensile strength after 10 kGy.	Amorphous material, more processable than PTFE.
PEEK	600 kGy	Dielectric alterations observed; mechanical properties maintained at high absorbed doses.	Used in high-performance applications.
DAP	10 kGy	Resistance maintained; used in radiation tracking experiments.	Insulating properties and high rigidity.
Vespel SP-1	100 MGy	8% loss in flexural strength at high absorbed doses; no change in thermal properties.	High thermal resistance; used in nuclear and aerospace applications.
Torlon 4203	100 Gy	Mechanical and thermal properties unchanged at low absorbed doses.	Similar resistance to Vespel SP-1; still limited in technical data.

**Table 2:** Descriptions of polymer applications and mechanisms/effects of irradiation on these polymers.

Section	Application	Mechanism	Ref.
Advanced Manufacturing	Additive Manufacturing	Radiation-induced crosslinking enhances mechanical integrity.	[32,103,110,111,112,113,114,115,116,117,118]
	Shape Memory Materials	Enables recovery of original form under specific stimuli.	[68,69,72,119,120,121,122,123,124,125,126,127,128]
Nuclear Industry	Cable Coatings	Enhanced thermal and radiation resistance for reactor cables.	[2,3,47,129,130,131]
	Radiation Shielding	Polymers that attenuate neutron radiation.	[132,133,134,135]
	Hydrogen Confinement	Specialized polymers prevent leakage in nuclear facilities.	[2,10,136,137,138]
Biomedical Applications	Medical Implants	Improved wear resistance and biocompatibility.	[139,140,141,142,143,144]
	Drug Delivery Systems	Enables controlled drug release via grafting.	[145,146,147]
	Sterilization	Ensures safety of medical devices without compromising properties.	[148,149,150,151,152]
Environmental and Energy Applications	Water Filtration	Enhanced membranes for desalination and wastewater treatment.	[153,154,155]
	Energy Storage	Polymers improve battery and supercapacitor performance.	[156,157,158]
	Photovoltaics	Optimized encapsulation materials for solar panels.	[159,160,161]
Consumer Goods	Packaging Materials	Improved toughness and thermal resistance for food packaging.	[2,162,163]
	Textiles	Enhanced fibers with increased strength and abrasion resistance.	[164,165,166,167,168,169]
Military and Aerospace	Spacecraft Components	Radiation-resistant polymers withstand extreme space conditions.	[170,171,172,173]
	Protective Coatings	Durable coatings for harsh environmental conditions.	[174,175,176]
	Ballistic Protection	Irradiation induces grafting, modifying chemical and mechanical properties, and enhancing polymer functionality.	[177,178,179]
Grafting	Monomers	Grafting monomers create new copolymers with properties tailored to the desired use.	[180,181,182,183,184]
	Molecules	The grafting of specific molecules can create polymeric compounds with advanced functionalities.	[48,185,186,187]
	Vulcanization	Combines sulfur grafting with crosslinking, offering significant structural improvements.	[71,188,189,190,191,192]
Surface Coating	Ink pigmentation	Ionizing radiation can degrade organic pigments and alter oxidation states of inorganic pigments.	[193,194,195,196]
Sustainability	Recycling	Ionizing radiation causes degradation and polymer chain scission, reducing molecular weight and facilitating chemical or mechanical recycling.	[197,198,199]

## Data Availability

Not applicable.

## Abbreviations

The following abbreviations are used in this manuscript:

ABS	Acrylonitrile–Butadiene–Styrene
CdCl_2_	Cadmium Chloride
COC	Cyclic Olefin Copolymer
CPG	Chitosan@PVA@rGO
CR-39	Polyallyldiglycol Carbonate
CS	Chitosan
DAP	Polydiallyl Phthalate
DSC	Differential Scanning Calorimetry
EIS	Electrochemical Impedance Spectroscopy
FEP	Fluorinated Ethylene Propylene
FP	Fluoropolymer
FT-IR	Fourier Transform Infrared Spectroscopy
HA	Hydroxyapatite
HIPS	High-Impact Polystyrene
LCP	Liquid Crystal Polymer
NIST	National Institute of Standards and Technology
PA6	Polyamide 6
PA9T	Poly(nonamethylene terephthalamide)
PA11	Polyamide 11
PA12	Polyamide 12
PA46	Polyamide 46
PA66	Polyamide 66
PBT	Poly(butylene terephthalate)
PC	Polycarbonate
PE	Polyethylene
PEI	Polyetherimide
PEO	Polyethylene Oxide
PEEK	Poly(ether-ether-ketone)
PES	Polyethersulfone
PET	Polyethylene Terephthalate
PI	Polyimide
PLA	Polylactic Acid
PMMA	Poly(methyl methacrylate)
PMP	Polymethylpentene
PP	Polypropylene
PPS	Polyphenylene Sulfide
PSC	Polymer Solar Cell
PS	Polystyrene
PTFE	Polytetrafluoroethylene
PVC	Polyvinyl Chloride
PVF	Poly(vinyl fluoride)
rGo	Reduced Graphene Oxide
Rct	Charge-Transfer Resistance
RTV-5370	Siloxane Rubber
SAN	Styrene–Acrylonitrile
$T_{g}$	Glass Transition Temperature
TGA	Thermogravimetric Analysis
TPA	Thermoplastic Amide
TPC	Thermoplastic Copolyester
TPU	Thermoplastic Polyurethane
TPO	Thermoplastic Polyolefin
TPS	Thermoplastic Styrene
TPV	Thermoplastic Vulcanizate
UHMWPE	Ultra-High-Molecular-Weight Polyethylene
XLPE	Crosslinked Polyethylene
XRD	X-ray Diffraction
